# Cooking Increased the Postprandial Glycaemic Response but Enhanced the Preload Effect of Air-Dried Jujube

**DOI:** 10.3390/foods14071142

**Published:** 2025-03-25

**Authors:** Jinjie Wei, Anshu Liu, Zhihong Fan, Xiyihe Peng, Xinling Lou, Xuejiao Lu, Jiahui Hu

**Affiliations:** 1 College of Food Science and Nutritional Engineering, China Agricultural University, 17 Qinghua East Road, Beijing 100083, China; 13312551374@163.com (J.W.); liuanshu@cau.edu.cn (A.L.); pxyh0831@cau.edu.cn (X.P.); 15208636232@163.com (X.L.); feirlu@163.com (X.L.); hujiahui1023@126.com (J.H.); 2 Key Laboratory of Precision Nutrition and Food Quality, Department of Nutrition and Health, China Agricultural University, Beijing 100193, China

**Keywords:** jujube, glycaemic response, insulin, preload, food matrix

## Abstract

Randomised controlled trials involving healthy participants were conducted to investigate the impact of cooking and ingestion patterns on the physiological response and preloading effect of air-dried jujube (AJ). The participants’ postprandial glycaemic and insulinemic responses were tested after ingestion of cooked or uncooked air-dried jujube containing 50 g (as a sole food source) or 15 g (as a preload food prior to a rice meal) of available carbohydrates. Compared with the uncooked AJ, the cooked air-dried jujube (CAJ) induced a 34.5% higher glycaemic peak, 57.1% greater glycaemic variability, and a 159.1% larger negative area under the glycaemic response curve when ingested as the only food in a meal. When eaten as a preload prior to a rice meal, the CAJ reduced the postprandial glycaemic peak by 25.17%. The CAJ preload enhanced insulin production in the 15 min after preloading but did not increase the total amount of postprandial insulin secretion. The result suggests that when taken as preload, the loose matrix of cooked fruits may exhibit glycaemic benefits by eliciting early insulin production and may therefore be conducive to the blood glucose management of a carbohydrate-laden meal.

## 1. Introduction

The increasing prevalence of diabetes and prediabetes is a serious public health issue in both developed and developing countries. Among the prediabetic population, impaired glucose tolerance often occurs when fasting glucose is still within the normal range [1]. Uncontrolled postprandial glycaemia is a manifestation of both poor metabolic status and increased risk of compromised pancreatic β-cell function [1]. In addition, impaired postprandial glucose and insulin homeostasis are strongly associated with a number of diseases, including polycystic ovary syndrome, obesity, and cardiovascular disease [2,3,4].

Meal pattern modification, such as pre-meal loading—an eating pattern in which a small amount of food (approximately one-quarter to one-third of the energy content of a regular meal) is consumed about 30 min before the main meal [5]—has been proposed as a potential dietary strategy to stabilise the postprandial glycaemic response. As pre-meal load foods, whey protein [6], milk and soymilk [7], kiwifruit, and tomato juice have been reported to mitigate postprandial glycaemic responses [8,9]. Even when provided as an extra carbohydrate load, fruit containing 15 g available carbohydrate (AC) ingested 30 min prior to a rice meal helped to reduce postprandial glycaemic variability [10].

Dried fruits are nutrient-dense and a good source of minerals, dietary fibres, and polyphenols. Previous studies found that dried fruits mostly have a low or medium glycaemic index (GI) and can be used as healthy substitutes for added sugar [11], and the GI of a meal containing mixed rice–dried fruits (25 g AC each) was lower than that of the rice meal [12]. Since dried fruits have previously been consumed with starchy food, in small amounts, their potential use as a preload food has been tested by an acute trial. On an isocarbohydrate basis, compared with the non-preloaded rice meal, preloading with dried apple containing 15 g AC before eating a rice meal resulted in a 28-unit reduction in the glycaemic index (GI) [13]. 

As one of the most consumed dried fruits in China, air-dried jujube (*Ziziphus jujube* Mill) is also used as an ingredient in traditional medicines. In recent years, in vitro studies and some animal experiments have suggested that bioactive compounds, including jujube polysaccharides [14], jujube pigments, and triterpenoids, might have the potential to improve blood glucose homeostasis [15,16]. However, it is unclear whether cooked dried fruits have a similar glycaemic effect, as cooking destroys the natural matrix of food and accelerates the release of sugars.

As air-dried jujube (AJ) is a common, widely consumed ingredient in pastry, rice, bread, congee, and soup, the characteristics of its glycaemic response after cooking must be considered. Previously, we found that stewed dried jujube elicited greater glycaemic variability and hypoglycaemic response than raw jujube, although the GI of stewed jujubes was comparable to that of uncooked dried jujubes [17]. When the dried jujube was cooked with rice (25 g AC each), the mixed porridge exhibited a significantly higher GI compared with the mixture of uncooked dried jujube and rice (85 vs. 75). However, the insulin response elicited by raw and cooked AJ was not measured in this study. 

Based on the results of the apple preload trial, we expected that the DJ might have a similar glycaemic effect when used as a preload food. However, it was possible that the raw and cooked AJ preload might differ in terms of the glycaemic and insulinaemic response, as the phenolic content, the viscosity of the matrix, and digestive properties would be modified during the cooking process. We hypothesised that the less viscous matrix, easier access to digestive enzymes, and the rapid release of sugar after cooking would promote the early production of insulin and thus enhance the preload effect.

In our randomised crossover human trial, AJ and cooked air-dried jujubes (CAJ) were tested as a single food (containing 50 g AC) or as a preload (containing 15 g AC) eaten prior to a rice meal, respectively. The postprandial glycaemic and insulinaemic responses were measured to compare the effects of the two arms, i.e., cooking treatment and intake patterns. In addition, changes in the textural properties, total phenolic and flavonoid content, in vitro digestive viscosity, and α-amylase and -glucosidase inhibition rates of air-dried jujube products before and after cooking were determined to explore the possible associations between the food treatment and postprandial physiological responses.

## 2. Materials and Methods

### 2.1. Materials and Instruments

Air-dried jujubes were made from grey jujubes (*Ziziphus jujuba* Mill.) harvested in October 2021 from Xinjiang, China, and processed by pitting, slicing, and then convection-dried at Henan Goodwill Co. (Zhengzhou, China). Rice (japonica type), at 5 kg, was provided by Fulinmen Brand, COFCO Group (Harbin, China). The determination of available carbohydrates was carried out by the Testing Center of China Agricultural University using ion chromatography. The other equipment and materials used are listed below:

A Human Insulin ELISA Kit from Shanghai Enzyme Link Biotechnology Co. (Shanghai, China); gallic acid, pepsin, pancreatic Enzyme, Forintol Reagent, pig bile salts, and ammonium carbonate from Beijing Solepol Science and Technology Co. (Beijing, China); (+)-Catechin, α-Nitrophenyl--D-Glucoside, acarbose hydrate, and calcium chloride from Shanghai McLean Biochemistry and Technology Co., Ltd. (Shanghai, China); soluble starch from Sinopharm Chemical Reagent Co. (Beijing, China).

MY-HT5093 Pressure Cooker from Meitai Life Electric Appliance Manufacturing Co., Ltd. (Guangzhou, China); ONE TOUCH UltraEasy Blood Glucose Meter and Test Strips from Johnson & Johnson Medical Devices Co., Ltd. (Xuancheng, China); TA.XT plus Physical Properties Tester from Stable Micro Systems, Ltd. (Surrey, UK); Synergy Multi-Functional Enzyme Labelling Instrument from Berten Instruments, Ltd. (Angleur, Belgium); Rotational Rheometer DHR-2 from TA Instruments, Ltd. (New Castle, DE, USA).

### 2.2. Measurement of Viscosity of Simulated Digested Chyme In Vitro

A three-stage in vitro digestion model that simulated the mouth, stomach and small intestine process was used [18]. Raw or cooked AJ containing 12 g AC were weighed and homogenised by equalisation to 80 g with deionised water. In the oral phase, 5 g of homogenate was mixed with 4.975 mL of simulated saliva and 25 μL calcium chloride solution (0.3 mol/L) and shaken at 200 rpm for 2 min at 37 °C. In the gastric phase, the digested samples from the oral phase were mixed with 4 mL of pepsin solution and 5 μL of calcium chloride solution, pH-adjusted to 3.0, and shaken at 37 °C for 2 h. In the small intestinal phase, the digested samples from the gastric phase were mixed with 8 mL of pancreatic enzyme solution, 40 μL of calcium chloride solution, and 0.6 g of bile salts. The pH was adjusted to 7.0 and the mixture was oscillated at 37 °C for 2 h. At the end of each digestion phase, an aliquot of the sample was carefully transferred onto the Peltier loading platform with a spatula. A 40 mm parallel plate fixture was employed, with the testing gap set at 1 mm. The measurement was conducted under controlled conditions at a temperature of 37 °C for a duration of 120 s. For samples from the oral stage, the shear rate was in the range of 0.001–50 s^−1^. For samples from the gastric and small intestine stages, the shear rate ranged from 0.001 to 30 s^−1^. Each sample was tested in quintuples [19]. A power law equation was used to establish the viscosity as a function of shear rate, as shown in Equation (1):

(1) $$\eta =K\times {\dot{\gamma }}^{n-1}$$

where η is the viscosity (Pas), K is the consistency coefficient (Pa.s^n^) (reflecting the viscosity of the fluid at a shear rate of 1 s^−1^), γ is the shear rate (s^−1^), and n is the rheological index (this reflects the fluid’s sensitivity to shear rate).

### 2.3. Determination of Total Phenol and Total Flavonoid Contents

We added 25 mL of 80% methanol solution to a 7.5 g homogenised sample, then ultrasonic extraction was performed for 15 min and the extracts were centrifuged at 8000 rpm for 10 min at 4 °C. After the supernatant was collected, the above steps were repeated for the precipitate (10 mL of methanol solution was added for the second time and 5 mL for the third time). The supernatants obtained from the three extraction procedures were combined and subsequently adjusted to a final volume of 50 mL with 80% methanol. The total phenolic content was determined by the Forintol method and expressed as gallic acid equivalents per 100 g dry weight (mg GAEs/100 g DW) [20]. The total flavonoid content was determined via the aluminium trichloride chromogenic method and expressed as catechin equivalents per 100 g dry weight (mg CAEs/100 g DW) [21].

### 2.4. Determination of α-Amylase and α-Glucosidase Inhibition Capacity

The experimental procedure was based on previous work by Zhang et al. and Li et al. [22,23]. Dried or cooked jujubes containing 2 g AC were weighed and homogenised by adjusting to 164.5 g with deionised water. To determine the α-amylase inhibition, 100 μL of homogenate was aspirated, then 100 μL of tryptic amylase solution (2 U/mL) was added, and the solution was maintained at a temperature of 37 °C for 10 min. Next, 100 μL of 1.5% soluble starch solution was added and maintained at 37 °C for 10 min, and 30 μL of the mixture was aspirated with 200 μL of DNS reagent, incubated in a water bath for 5 min, and rapidly cooled on ice; the absorbance measured at 540 nm. The positive control was 0.05 mg/mL acarbose solution. To measure the α-glucosidase inhibition, 100 μL of homogenate was aspirated and 100 μL of α-glucosidase solution (0.5 U/mL) was added and maintained at a temperature of 37 °C for 10 min. Then, 100 μL of 6 mM α-nitrophenyl-D-glucoside solution was added and maintained at 37 °C for 10 min; finally, 200 μL of 1M sodium carbonate solution was added and the absorbance was 405 nm. The positive control was 0.05 μg/mL acarbose solution. The α-amylase and α-glucoamylase inhibition rates were calculated according to Equation (2):

(2) $$\mathrm{Inhibition}\;\mathrm{rates}/\%=(1-\frac{A_{sample}-A_{blank}}{A_{control}})\times 100$$

where A_sample_ is the absorbance of the reaction solution of the sample group; A_blank_ is the absorbance of the enzyme solution replaced with phosphate buffer; A_control_ is the absorbance of the sample replaced with phosphate buffer.

### 2.5. Participants

Healthy participants aged 18–26 years with a body mass index (BMI) of 18.5–23.9 kg/m^2^ were recruited via the social platform of the campus of China Agricultural University (CAU). The exclusion criteria were as follows: having a diagnosed metabolic or digestive disorder; having been on a diet or lost weight within the past 3 months; frequently eating late at night or skipping breakfast; being allergic to the foods used in the experiment; smoking and drinking; engaging in high-intensity sports training; suffering from eating disorders; and taking medications that would affect digestion or metabolism. Participants who did not meet any of the exclusion criteria underwent an oral glucose tolerance test (OGTT). The inclusion criteria were a fasting blood glucose of less than 6.0 mmol/L, a postprandial blood glucose peak of less than 11.1 mmol/L, and a 2-h postprandial blood glucose of less than 7.8 mmol/L. Those who did not meet the criteria were screened. This trial was approved by the Human Research Ethics Committee of China Agricultural University (Ethics No. 20220201) and registered with the China Clinical Trial Registry (Registration No. ChiCTR2200057371).

The blood glucose and insulin response tests and the preprandial loading trials with dried jujubes and cooked dried jujubes were conducted according to a controlled randomised crossover design, in which the participants were allocated to test sessions in a random, computer-generated order, with a minimum of a 3-day washout between test sessions. The day before each test, the participants were asked to eat an identical dinner in the school cafeteria, not to drink coffee or alcoholic beverages, not to overeat, not to stay up late, and to avoid high-intensity training. After 12 h of overnight fasting, they entered the laboratory at 8 am on the test day.

### 2.6. Test Meal and Trial Procedures

#### 2.6.1. Blood Glucose and Insulin Response of AJ and CAJ

The test meals each consisted of 50 g of AC, including (1) a glucose solution (G) comprising 55 g of glucose monohydrate (C_6_H_12_O_6_.H_2_O) dissolved in 250 g of water; (2) 69.9 g air-dried jujube (AJ) and 250 mL water; and (3) cooked air-dried jujube (CAJ) prepared by mixing 69.9 g AJ and 250 g of deionised water followed by 7 min of heating in an electric pressure cooker at 115 °C. This cooking method was pre-tested to ensure the complete destruction of the natural structure of the food. The volume of the test meals was balanced by drinking water. 

**Figure 1:**
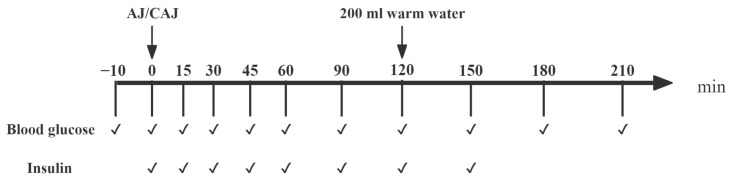
The procedure of the trials examining postprandial glycaemic and insulinaemic responses after consuming raw and cooked air-dried jujubes. Notes: “✔” means at the current time point, blood glucose testing or blood sample collection (for insulin analysis) are required.

The test protocol is shown in Figure 1. Duplicated fasting fingertip blood samples were collected (−10 and 0 min) before the test meal was consumed. The meal was completed within 15 min and the blood samples were obtained at fixed time points. Blood glucose was measured using a glucometer and the insulin concentration was determined using an ELISA kit (JunLB Ltd., Beijing, China).

#### 2.6.2. Blood Glucose and Insulin Responses to Rice Meal with AJ and CAJ as Preload

The test meals, each containing 50 g of AC included (1) dried jujube preload containing 15 g AC and rice containing 35 g AC (AJ+R); (2) cooked dried jujube preload containing 15 g AC and rice containing 35 g AC (CAJ+R); and (3) water preload and rice (W+R) consisting of 66.1 g of raw rice and 132.2 g of distilled water. The specific composition of the test meals is shown in Table 1. The cooking process of the dried jujube is described in Section 2.6.1. The rice (66.1 g of raw rice and 132.2 g of distilled water) was cooked with a rice cooker on ‘steaming’ mode for 30 min.

**Figure 2:**
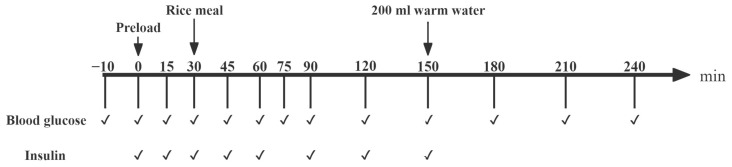
The procedure for the preload trial study. Notes: “✔” means at the current time point, blood glucose testing or blood sample collection (for insulin analysis) are required.

The protocol for the preload trials is shown in Figure 2. After the duplication of the fasting fingertip blood samples (−10 min and 0 min), the preload food was provided and consumed within 10 min. A rice meal was provided 30 min after the preload and this meal was completed within 10 min. Blood was collected via a finger prick test and the blood glucose and insulin level were measured at fixed time points, as described in Section 2.6.1.

### 2.7. Statistical Analyses

The sample size was calculated using PASS 2021 (NCSS, Kaysville, UT, USA). Based on the results of Zhu et al. [12,17], 10 participants could provide 80% power to test for differences in postprandial blood glucose peaks (*p* < 0.05).

The increases in postprandial glucose and insulin were calculated by subtracting the fasting glucose or insulin value from the same value at each postprandial time point. 

The blood glucose and insulin change values were acquired by subtracting the fasting blood glucose and fasting insulin values from the postprandial blood glucose and postprandial insulin values at each postprandial time point. The quadrilateral method was used to calculate the incremental area under the curve (iAUC_glu_ and iAUC_ins_), the postprandial response over the entire experimental period of 0–120 min, and the negative area under the curve (NAUC_glu_). NAUC_glu_ is the area enclosed by the portion of the curve that lies below the baseline (usually the zero line or reference line) during a specific measurement or analysis process, and it may be a better predictor of postprandial self-reported hunger and subsequent energy intake than peak glucose at 0–2 h and the glucose incremental area under the blood glucose curve at 0–2 h [24]. The iAUC during 0–60 min, 0–30 min and 30–90 min periods (iAUC_glu0–60%_ and iAUC_ins0–60%_, iAUC_glu0–30%_ and iAUC_ins0–30%_, iAUC_glu30–90%_ and iAUC_ins30–90%_) were calculated as the percentages of the total glucose or insulin AUC from 0 to 180 min (iAUC_glu0–180_ or iAUC_ins0–180_), respectively. The peak blood glucose and insulin changes (PEAK_glu_ and PEAK_ins_) and time to peak were recorded. Consecutive 1 h intervals of net glucose/insulin action (CONGA1_glu_ and CONIA1_ins_) were used to measure fluctuations in glucose or insulin [25]. The area under the postprandial insulin resistance index curve (the area under the HOMA-IR curve, HOMA-IR AUC) was calculated throughout the trial to measure postprandial insulin sensitivity [26]. Based on the iAUC_glu0–120_ and iAUC_ins0–120_ values, the glycaemic index (GI), defined as 100 × iAUC_glu0–120 sample_/iAUC_glu0–120 control_ and insulinaemic index (II), defined as 100 × iAUC_ins0–120 sample_/iAUC_ins0–120 control_, were calculated using the glucose solution as the reference food [27]. 

A linear mixed-effects model (LMEM) was used to test for between-group differences in postprandial glucose and insulin change values and eigenvalues. The physicochemical indices of the samples were analysed by one-way ANOVA or Kruskal–Wallis test, with differences in the viscosity of sample digests analysed by a general linear model. Multiple comparisons were corrected for *p*-values using the LSD method, and *p* < 0.05 was considered to be a significant difference. Statistical analyses were performed with IBM SPSS Statistics 27.0.

## 3. Results

### 3.1. Total Phenol and Flavonoid Contents

**Figure 3:**
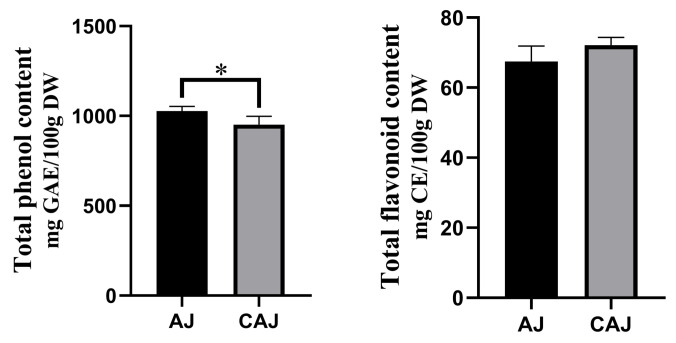
The total phenolic and flavonoid contents of air-dried jujube and cooked air-dried jujube. Note: * indicates that there is a significant difference between air-dried jujubes and cooked dried jujubes, *p* < 0.05.

The total phenolic and flavonoid contents of AJ and CAJ are shown in Figure 3. The total phenolic content of AJ was slightly higher than that of CAJ, while there was no significant difference in the total flavonoid content.

### 3.2. α-Amylase and α-Glucosidase Inhibitory Activity

**Figure 4:**
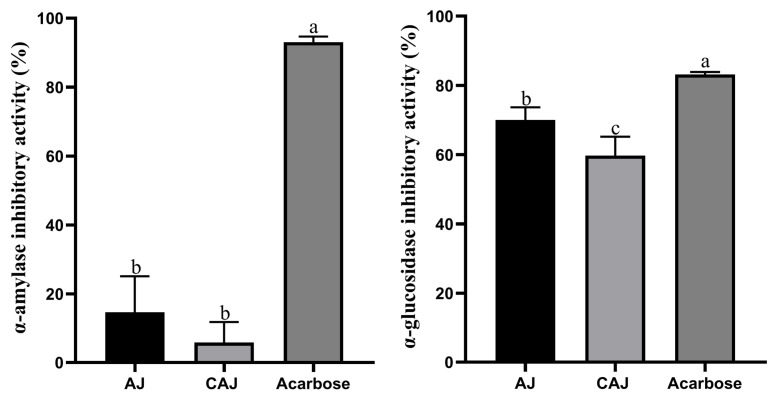
α-amylase and α-glucosidase inhibitory activity. Note: “a, b, c” are used to indicate comparisons of differences between groups.

The α-amylase and α-glucosidase inhibition activity of dried jujube before and after cooking is shown in Figure 4. The α-amylase inhibition activity of both AJ and CAJ was low, exhibiting no significant difference. In contrast, the inhibition activity of α-glucosidase of AJ was slightly higher than that of the CAJ, though both were relatively high.

### 3.3. In Vitro Digest Viscosity

The apparent viscosity of all the samples tested decreased as the shear rate increased, exhibiting shear-thinning characteristics. 

**Figure 5:**
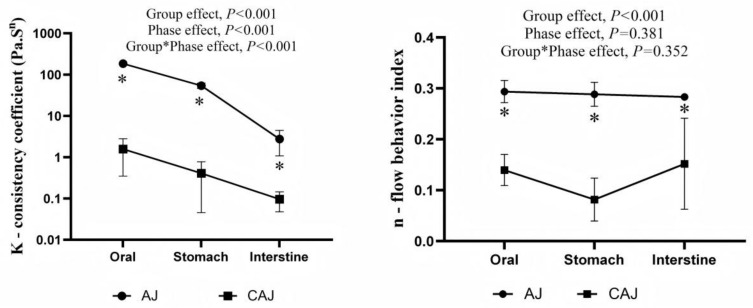
Power law parameters of the different digesta of air-dried jujube and cooked air-dried jujube. Note: * indicates that there is a significant difference between air-dried jujubes and cooked dried jujubes, *p* < 0.05.

The model fit was good, with correlation coefficients (R^2^) close to 1 (0.9–0.99) for all samples. The correlation parameters of the power law equation for each sample are presented in Figure 5. The consistency coefficients and rheological indices of the AJ in in vitro digesta at each stage were significantly higher than those of the CAJ, indicative of the higher viscosity and shear stability.

### 3.4. Participant Characteristics

**Figure A1:**
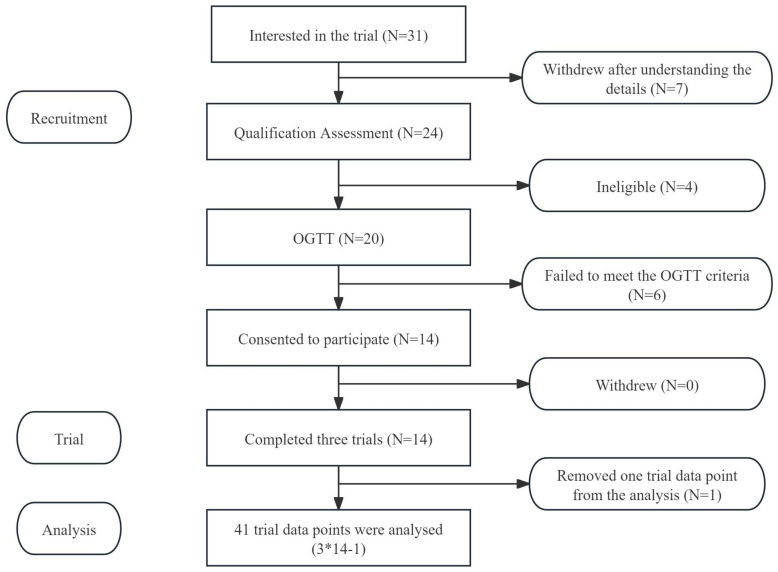
Flow diagram of the study subjects from the glucose and insulin response trial. Notes: “*” means multiplication, “-” means subtraction.

**Figure A2:**
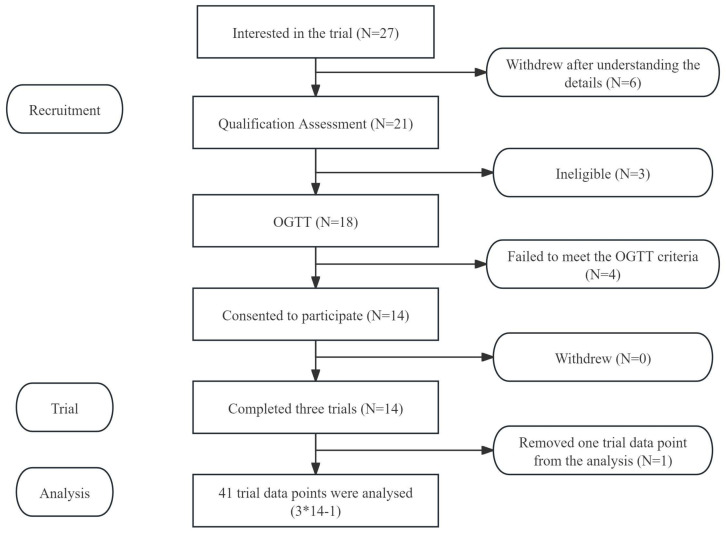
Flow diagram of the study subjects from the preload trial. Notes: “*” means multiplication, “-” means subtraction.

The glucose and insulin response trial and preload trial involved two distinct groups of participants. The glycaemic and insulin response tests of AJ and CAJ included 14 participants (6 males and 8 females), and the preload trial also included 14 participants (7 males and 7 females). Basic information about the participants is provided in Table 2. All participants completed the scheduled trials and none of them reported any discomfort after consuming the test meals. Only 13 participants’ insulin data were included in the final analysis because one of the blood samples was affected by haemolysis. Flow diagrams of the study participants are shown in Figure A1 and Figure A2.

### 3.5. Effect of Cooking on the Postprandial Physiological Response of Air-Dried Jujube

**Figure 6:**
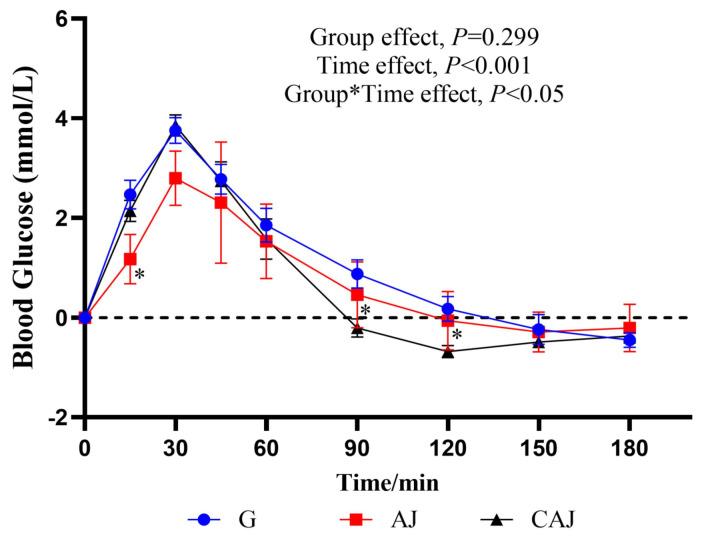
The postprandial glycaemic response curves after ingesting raw and cooked air-dried jujubes. Note: * denotes significant difference between raw and cooked air-dried jujubes, *p* < 0.05. Group*Time means interaction effect between group and time.

The postprandial blood glucose response curves following ingestion of AJ and CAJ are shown in Figure 6. The 15 min blood glucose after ingesting the CAJ was higher than that of the AJ, and the 30 min blood glucose tended towards being higher than that of the AJ (*p* = 0.085), indicating that CAJ caused a faster rise in blood glucose than air-dried jujube in the first 30 min. The 90 and 120 min blood glucose after ingesting the CAJ was significantly lower than that of the AJ, while the 180 min blood glucose after ingesting the CAJ tended to be lower than that of the AJ (*p* = 0.075). The postprandial glycaemic response after ingesting the CAJ showed a dramatic glucose surge and a steep post-peak decline over a period of 45–120 min. In contrast, the response after ingesting the AJ showed a comparatively stable glycaemic pattern. 

The postprandial glycaemic responses to each sample are listed in Table 3. Compared with the AJ, the CAJ had a 159.1% larger NAUC_glu_, a 34.5% higher PEAK_glu_ and a 57.1% elevated CONGA1_glu_, suggesting that the CAJ elicited greater postprandial glycaemic variability, including a higher risk of hyperglycaemia at 0–60 min and a greater risk of hypoglycaemia at 90–180 min. Despite the remarkable differences in the postprandial glycaemic pattern, the difference between the AJ and CAJ was insignificant in terms of GI.

**Figure 7:**
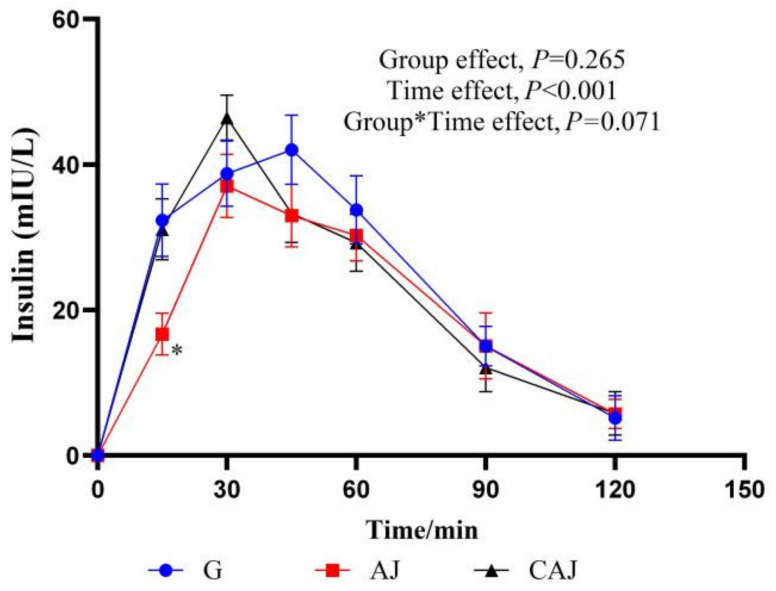
The postprandial insulinemic response curves of raw and cooked air-dried jujubes. Note: * denotes significant difference between raw and cooked air-dried jujubes, *p* < 0.05. Group*Time means interaction effect between group and time.

The insulin response curves after AJ and CAJ consumption are shown in Figure 7. In the CAJ group, the insulin value recorded at 15 min was significantly higher than that of the AJ group, and the insulin value obtained at 30 min tended towards being higher than that of the AJ group (*p* = 0.071), suggesting that CAJ induces insulin secretion more effectively in the early stages of feeding.

The characteristics of the postprandial insulin response are shown in Table 4. Higher PEAK_ins_ was observed in the CAJ group than in the AJ group (*p* = 0.053). The HOMA-IR AUC of the CAJ tended to be higher than that of the AJ (*p* = 0.061), highlighting a tendency towards decreased postprandial insulin sensitivity after the consumption of CAJ. However, there was no significant difference in the insulin index between the two groups.

### 3.6. Impact of Cooking on the Preloading Effect of Air-Dried Jujube

**Figure 8:**
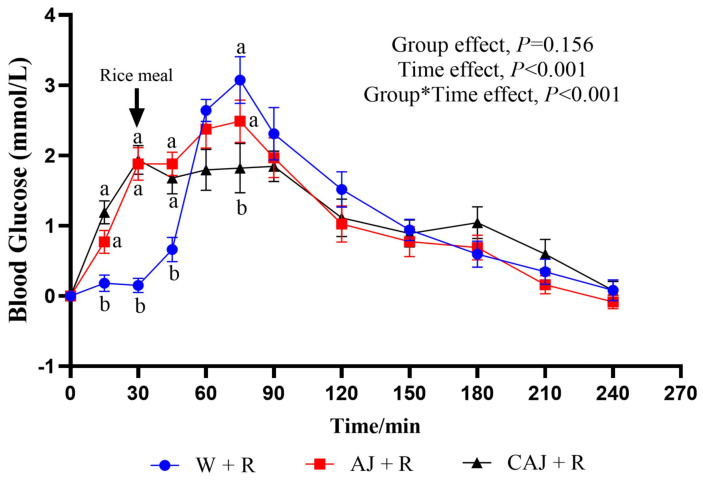
The postprandial glycemic response curves of preload test meals. AJ+R: air-dried preload and rice; CAJ+R: cooked air-dried preload and rice; W+R: water preload and rice as control. Note: “a, b” are used to indicate comparisons of differences between groups. Group*Time means interaction effect between group and time.

The glycaemic response curves after consumption of the two preloads and rice meals are shown in Figure 8. The glucose level peaked at 75 min for both the AJ+R group and the W+R group, while it peaked at 30 min in the CAJ+R group. At the 30 min time point, blood glucose levels were significantly higher in the AJ+R and CAJ+R groups than in the W+R group. The CAJ preload group tended to have a higher glucose level than the AJ preload group at the 15 min time point (*p* = 0.096). After the consumption of the rice meal, the blood glucose level of the CAJ+R group showed no increase during the 45–90 min interval. A tendency towards a lower blood glucose level was observed at 75 min in the CAJ+R group compared with the AJ+R group (*p* = 0.067). 

The postprandial glycaemic response parameters are listed in Table 5. The iAUC_glu0–30_ of the AJ+R and CAJ+R groups was significantly higher than that of the W+R group, indicating that the preload-induced increase in blood glucose accounted for a large proportion of the total rise in blood glucose. The iAUC_glu30–90_ in the CAJ+R group was significantly lower than that in the AJ+R group and tended to be lower than that in the W+R (*p* = 0.055). In addition, only the CAJ+R group had significantly lower PEAK_glu_ than the W+R group, exhibiting a 25.17% reduction. Compared with the control group, the CAJ+R and AJ+R groups exhibited 36.8% and 21.1% reductions in CONGA1_glu_, respectively. The CAJ+R tended to be lower than the AJ+R preload group in terms of CONGA1_glu_ (*p* = 0.077), which was indicative of better postprandial glycaemic stability.

**Figure 9:**
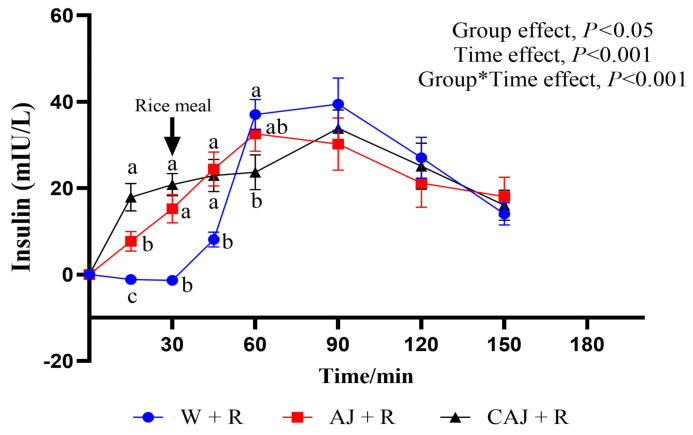
The postprandial insulinemic response curves of the preload test meals. AJ+R: air-dried preload and rice; CAJ+R: cooked air-dried preload and rice; W+R: water preload and rice as control. Note: “a, b, c” are used to indicate comparisons of differences between groups. Group*Time means interaction effect between group and time.

The insulin response curves resulting from the test meals are shown in Figure 9. The insulin levels increased significantly after preloading, with peak values occurring at 90 min in the CAJ+R and W+R groups and at 60 min in the AJ+R group. CAJ+R had a significantly higher insulin level than AJ+R and W+R at 15 min but exhibited a lower level at 60 min compared with W+R (*p* < 0.05) and AJ+R (*p* = 0.071).

The parameters of the postprandial insulin response are shown in Table 6. There was no significant difference in iAUC_ins_ among the three test groups. The iAUC_ins0–30_ were significantly higher in the CAJ+R group, suggesting that ingesting CAJ elicited significant insulin secretion prior to rice consumption. The iAUC_ins30–90_ were lower in the CAJ+R group than in the AJ+R group and tended to be lower than in the W+R group (*p* = 0.068). The CONIA1_ins_ of the AJ+R and CAJ+R groups were 23.22% and 34.2% lower, respectively, than that of the W+R group. No significant difference in iAUC_ins_ and HOMA-IR AUC was observed among the three sample groups.

## 4. Discussion

This study investigated the glycaemic properties of uncooked and cooked air-dried jujubes either as the sole carbohydrate source or as a preload eaten prior to a rice meal. When consumed on its own, the cooked sample induced a dramatic postprandial blood glucose excursion; however, when a small amount was consumed prior to a high-GI rice meal, it provided a remarkable preload effect and minimised the glycaemic excursion that followed the main meal. 

The GIs of AJ and CAJ were 68 and 83, respectively, leading them to be classified as medium-and high-GI foods [28], respectively. The lack of significant difference between the two GI values can be explained by the high inter-individual variability, as well as the small sample size. Compared with the AJ, the CAJ induced a significantly higher peak blood glucose value, a larger magnitude of blood glucose fluctuation, and a greater negative area under the blood glucose curve. This suggests that heavy consumption of soft-boiled dried fruit products in one sitting may not be conducive to glycaemic homeostasis [16]. 

The GI of the CAJ in our study was much higher than the GI of the stewed jujube in our previous study (83 vs. 56) [16]. In addition to differences in the variety and origin of the jujube material, this may be because, in the present study, the air-dried jujube was sliced and cooked in a pressure cooker. Compared with the stewed jujube in the previous study, which was softened but still retained its shape, the complete destruction of the natural texture of CAJ promoted a more rapid release of sugar [29]. 

Dried fruits usually have a medium-to-low GI [11]. Their cohesive natural texture, polyphenol content, and dietary fibres can reduce the rate of sugar release and inhibit its absorption in the human body [30]. However, in the present study, despite the differences in the glycaemic behaviours between AJ and CAJ, their polyphenol content and enzyme-inhibiting capacity were comparable, and the fibre content was unaffected by the cooking process. Therefore, the noticeable difference between AJ and CAJ is more likely to be a result of their disparity in texture. At all digestive stages, the viscosity and shear stability of the digesta of CAJ were significantly lower than that of AJ, which was indicative of the severe tissue destruction in CAJ. Previous studies have shown that the viscosity of in vitro food chyme is negatively correlated with its postprandial glucose and insulin responses [31] as the high viscosity could delay the rate of gastric emptying, decrease the digestive enzymes’ access to food particles, and slow the rate of transportation and absorption of nutrients within the gastrointestinal tract [32]. Therefore, sugars in CAJ could be released and absorbed much more quickly than those in AJ, resulting in rapid stimulation of insulin production [33,34]. This mechanism is consistent with the surge in the insulin level of CAJ, which occurred 15 min after ingestion, mirroring the glucose reference.

However, when the CAJ containing 15 g AC was utilised as a preload prior to consuming rice (CAJ+R), it achieved better glycaemic variability. The insulin level of CAJ+R was significantly higher than that of AJ+R 15 min after ingestion, indicating that CAJ promoted insulin secretion more effectively than AJ. It is obvious that the insulin recruited by the preprandial load of CAJ was ready to address the glucose rise elicited by rice ingestion, preventing a sharp blood glucose increase. An earlier insulin response is believed to be beneficial in inhibiting a blood glucose spike and maintaining postprandial glycaemic stability [6]. 

In spite of the early rise in insulin levels, the total AUC and peak insulin level of the preload groups did not exceed that of the W+R. Furthermore, the combination of insulin AUC in 0–30 min and 30–60 min resulted in no significant difference among the three test meals. Since prediabetic patients with impaired glucose tolerance often suffer from reduced or lost early-phase insulin secretion [35], an efficient insulin mobiliser such as CAJ preload could represent a simple, easy-to-use countermeasure which does not place extra burden on pancreatic islet cells. 

Our results suggest that the preload effect of carbohydrate food may not be positively correlated with its GI value. Many nutrient-dense, high-GI foods do not need to be excluded from the diets of prediabetic or diabetic patients. When consumed in small amounts as a preprandial load, they may help to prevent a rapid rise in blood glucose after high-GI-carbohydrate consumption by promoting an early insulin response. 

In addition to sugar components, dried jujube is rich in phytochemicals such as triterpenoids, nucleosides, cyclic nucleotides, and bioactive polysaccharides and phenols, including flavonols, flavan-3-ols, hydroxybenzoic acids, and hydroxycinnamic acids [36]. Phenolic compounds improved postprandial glycaemia by delaying intestinal glucose transport and inhibiting α-amylase activity [37,38], determining the digestion rate of starchy foods [39]. 

We found that the loss of total phenolics and total flavonoids after cooking was rather minor in dried jujube, which was inconsistent with previous reports that the fruit polyphenols could remain well preserved after dehydration and cooking [40,41]. On the one hand, cooking treatments can promote the release of bound phenolics in material with a cellular structure and can increase extraction [42]. On the other hand, heating inhibits or inactivates oxidative enzymes and reduces oxidative losses [43]. In addition, the airtight environment within the pressure cooker may favour the preservation of polyphenols. However, the results of this study showed that the inhibition of α-amylase by both AJ and CAJ was low and not significantly different. This suggests that the inhibition of starch digestion by phenolics is unlikely to play a major role in the hypoglycaemic effects of the dried jujube preload meals.

Dietary fibre in fruits has been reported to inhibit postprandial hyperglycaemia by delaying gastric emptying, forming a gel in the intestine that can slow the absorption of glucose [44], and inducing the secretion of glucagon-like peptide (GLP-1) [45]. However, when AJ and CAJ were used as preload, the amount of dietary fibre was too limited to effectively suppress the digestion of the rice meal. Caloric load has a greater effect on gut hormone release than fibre [46], and incretins such as GLP-1 are only effective in inducing insulin secretion in response to elevated blood glucose levels [47]. Therefore, compared with sugar alone, the combination of sugar and fibre in the preprandial load of fruits or dried fruits may be better at promoting hormone release and helping regulate the postprandial glycaemic response. Zhao et al. found that a combination of starchy food and fibre as a preprandial load managed the postprandial glycaemic response more effectively than either partially hydrolysed guar fibre or potatoes alone when consumed 30 min prior to a meal [48]. 

The presence of organic acid in fruits may play a role, as they can downregulate the glycaemic response by inhibiting saliva amylase [49]. However, the jujube is a low-acidity fruit. Furthermore, the acidity of fruit food is less impacted by boiling when the amount of water is limited, as the ionisation of organic acids increases when they are diluted. 

Therefore, we suppose that the quick release of sugar resulting from the destruction of natural textures is the most likely explanation for CAJ’s superior preload effect compared to AJ. There is accumulating evidence that the matrix of foods is an important determinant of the metabolic effects both in the short and the long term [50]. Food preparation not only alters the food’s nutritional value but also its physiological effects after ingestion by modifying the rate of release of nutrients and the accessibility of digestive enzymes [51,52]. The results of this study suggest that modifying a food’s texture can influence the glycaemic response when combined with a proper meal pattern, even on the basis of identical chemical composition.

To the best of our knowledge, the present study is the first trial to compare the glycaemic effects of the same food when consumed either on its own as a sole carbohydrate source (50 g AC) or as a preload food (15 g AC) prior to a rice meal. We investigated the insulin pattern associated with the test meals and determined that the insulin-mobilising behaviour of the food might be the underlying mechanism responsible for the preload effect. In vitro assays help to explore the possible contributions of texture factors and the enzyme-inhibiting aspects of polyphenols. 

However, the present study has several limitations. Firstly, the trial only included healthy adults and should be further validated in other populations, such as prediabetics, diabetics, and people with impaired digestive function. Secondly, this study only investigated the acute effects of air-dried and cooked air-dried jujube on glycaemic responses. The glycaemic effects of long-term consumption of AJ or CAJ as preload foods have yet to be investigated. Finally, the effect of processing methods on the microstructure of air-dried jujube, as well as the possible contributions of phytochemicals other than polyphenols, were not investigated in this work.

## 5. Conclusions

In this study, we found that cooked air-dried jujube containing 50 g AC induced greater postprandial glycaemic variability than its uncooked counterpart when ingested in a single sitting. This result is likely to be related to the disruption of the natural texture and lowered viscosity of the coeliac as opposed to the enzyme-inhibiting capacity of the polyphenols in jujube. When ingested as a preprandial load containing 15 g AC, compared with its uncooked counterpart, the CAJ achieved a more successful reduction in both the blood glucose peak and the maximum glycaemic excursion after rice meal ingestion. This may be attributed to the fact that the CAJ efficiently induced early insulin recruitment without increasing total insulin secretion. 

Our results indicate that texture-modifying food preparation may play an important role with respect to the glycaemic properties of natural foods. Nutrient-dense, high-GI carbohydrates might be introduced for glycaemic management in the form of preload food ingested in small amounts. Given the large population of people with impaired glucose tolerance who are in need of postprandial glycaemic management, the long-term effect of using dried jujube and other dried fruits as a preprandial load deserves to be verified in future intervention studies. 

## Figures and Tables

**Table 1:** Ingredients and nutrient composition of test meal (per serving).

	Preload						Rice Meal	
	Food (g)	Water (g)	AC (g)	Glucose ^b^ (g)	Sucrose (g) ^b^	Fructose (g) ^b^	Rice (g)	AC (g)
AJ+R	20.96	116.04	15	2.2	2.2	6.0	69.9	35
CAJ+R	78.21	58.79	15	2.2	2.2	6.0	69.9	35
W+R	-	137	-	-	-	-	171.5 ^a^	50

(^a^) Rice weights (cooked); (^b^) monosaccharides were determined by ion chromatography.

**Table 2:** Participants’ baseline information.

	Glucose and Insulin Response Test (*n* = 14)	Pre-Meal Load Test (*n* = 14)
Age	25 ± 2	23 ± 2
BMI (kg/m^2^)	21.4 ± 1.6	22.1 ± 2.2
Body fat (%)	21.4 ± 5.0	22.9 ± 5.6
Waist (cm)	73.6 ± 7.8	75.3 ± 8.3
Hip measurement (cm)	92.8 ± 4.6	94.5 ± 5.3
Hip–waist ratio (%)	84.9 ± 3.9	79.6 ± 6.6
Basal metabolic rate (kcal)	1391.6 ± 217.0	1460.0 ± 198.6

**Table 3:** The postprandial glycemic parameters of air-dried jujube and cooked air-dried jujube.

Test Meal	iAUC_glu_ (mmol × min/L)	iAUC_glu0–60%_	NAUC_glu_ (mmol × min/L)	Peak_glu_ (mmol/L)	CONGA1_glu_ (mmol/L)	Glycemic Index
AJ	144.1 ± 12.6	70.7 ± 3.832 *	11.0 ± 3.7 **	2.9 ± 0.2 **	1.4 ± 0.61 **	68.0 ± 6.7
CAJ	171.0 ± 10.2	71.9 ± 3.348	51.3 ± 3.4	3.9 ± 0.1	2.2 ± 0.6	83.0 ± 5.6
G	226.9 ± 21.1	69.3 ± 4.493	32.4 ± 6.5	4.0 ± 0.2	1.9 ± 0.9	100

Note: * indicates that there is a significant difference between air-dried jujubes and cooked dried jujubes, *p* < 0.05, and ** indicates that the difference is highly significant, *p* < 0.001.

**Table 4:** The postprandial insulinemic parameters of air-dried jujubes and cooked air-dried jujubes.

Test Meal	iAUC_ins_ (mIU × min/L)	iAUC_ins0–60%_	HOMA-IR AUC (mmol × mIU\× min/L^2^)	Peak_ins_ (mIU/L)	CONIA1_ins_ (mIU/L)	Insulin Index
AJ	2520.6 ± 299.5	63.2 ± 3.1	1050.6 ± 147.7	37.1 ± 4.3	26.5 ± 9.7	88.0 ± 9.9
CAJ	2781.9 ± 246.8	70.0 ± 3.8	1214.1 ± 142.5	46.4 ± 3.1	29.3 ± 9.5	94.9 ± 6.7
G	2998.4 ± 208.4	65.7 ± 3.1	1329.6 ± 120.2	42.0 ± 4.4	30.1 ± 13.2	100

**Table 5:** The postprandial glycemic parameters of preload test meals. AJ+R: air-dried preload and rice; CAJ+R: cooked air-dried preload and rice; W+R: water preload and rice as control.

Test Meal	iAUC_glu_ (mmol × min/L)	iAUC_glu0–30%_	IAUC_glu30–90%_	Peak_glu_ (mmol/L)	CONGA1_glu_ (mmol/L)	Peak Time (min)
AJ+R	270.9 ± 28.2	10.2 ± 1.4 ^a^	49.9 ± 2.5 ^a^	3.1 ± 0.2 ^a^	1.5 ± 0.1 ^b^	61.0 ± 14.1
CAJ+R	282.4 ± 31.7	12.6 ± 1.1 ^a^	38.8 ± 3.3 ^b^	2.5 ± 0.2 ^b^	1.2 ± 0.1 ^b^	64.0 ± 34.0
W+R	264.5 ± 32.6	2.1 ± 0.6 ^b^	46.3 ± 2.3 ^ab^	3.4 ± 0.3 ^a^	1.9 ± 0.2 ^a^	71.0 ± 9.2

Note: “^a, b^” are used to indicate comparisons of differences between groups.

**Table 6:** The postprandial insulinemic parameters of preload test meals.

Test Meal	iAUC_ins_ (mIU × min/L)	iAUC_ins0–30%_	iAUC_ins30–90%_	HOMA-IR AUC (mmol × mIU\× min/L^2^)	Peak_ins_ (mIU/L)	CONIA1_ins_ (mIU/L)
AJ+R	3262.2 ± 509.2	7.0 ± 1.1 ^b^	53.0 ± 3.0 ^a^	1421.7 ± 136.5	41.4 ± 4.9	23.8 ± 2.6 ^b^
CAJ+R	3470.6 ± 317.3	13.2 ± 1.8 ^a^	46.2 ± 2.5 ^b^	1358.0 ± 140.7	43.6 ± 3.8	20.4 ± 2.4 ^b^
W+R	3228.1 ± 391.2	0.4 ± 0.1 ^c^	50.7 ± 2.8 ^ab^	1329.7 ± 130.6	46.8 ± 4.0	31.0 ± 2.9 ^a^

Note: “^a, b, c^” are used to indicate comparisons of differences between groups.

## Data Availability

The datasets presented in this article are not readily available due to privacy. Requests to access the datasets should be directed to the corresponding author.

## Abbreviations

The following abbreviations are used in this manuscript:

AC	Available carbohydrate
GI	Glycaemic index
AJ	Air-dried jujube
CAJ	Cooked air-dried jujube
OGTT	Oral glucose tolerance test
AJ+R	Preprandial load of dried jujubes and rice
CAJ+R	Preprandial load of cooked dried jujubes and rice
W+R	Preprandial load of water and rice
IAUC	Incremental area under the curve
NAUC	Negative area under the curve
CONGA1glu and CONIA1ins	Consecutive 1 h intervals of net glucose/insulin action
